# Molecular investigation in children candidates and submitted to cochlear implantation

**DOI:** 10.1016/S1808-8694(15)30965-4

**Published:** 2015-10-19

**Authors:** Raquel Bernardes, Silvana Bortoncello, Thalita Vitachi Christiani, Edi Lúcia Sartorato, Rodrigo César e Silva, Paulo R. Cantanhede Porto

**Affiliations:** a Speech and Hearing Specialist, Cochlear Implant Program – Department of Otorhinolaryngology/Head and Neck Surgery - UNICAMP; b Speech and Hearing Specialist, Cochlear Implant Program – Department of Otorhinolaryngology/Head and Neck Surgery - UNICAMP; c MS. Human Genetics Lab - CBMEG-UNICAMP; d PhD. Professor – Human Genetics Lab - CBMEG-UNICAMP; e MD. Resident of Otorhinolaryngology / Head and Neck Surgery - UNICAMP; f Head of the Cochlear Implant Program – Department of Otorhinolaryngology/Head and Neck Surgery - UNICAMP

**Keywords:** connexin 26, cochlear implants, sensorineural hearing loss

## Abstract

**Aim:**

recent progresses in molecular biology have been made in the diagnosis of sensorineural hearing loss. The high prevalence of a connexin 26 gene mutation, and its easy identification have made the diagnosis possible. The most frequent gene mutation is called 35delG. The purpose of this study was to evaluate the prevalence of 35delG mutation in children submitted to cochlear implantation who had severe and profound hearing loss previously diagnosed as idiopathic.

**Method:**

The study was done at the Cochlear Implantation Clinic of the Otolaryngology Department and at the Laboratório Genética Humana-CBMEG, UNICAMP-SP. 32 children with severe to profound sensorineural hearing loss were evaluated. The detection of the 35delG mutation was made by a allele - specific PCR, using primers and polymerase chain reaction.

**Results:**

69% had a normal exam, 12% were homozygous for the mutation, 19% of the cases were heterozygous. The 35delG mutation in heterozygousity is not a cause of hearing loss.

**Conclusion:**

The data confirm the high prevalence of the 35delG mutation in nonsyndromic bilateral profound sensorineural hearing loss. It was also possible to diagnose the cause of hearing loss as genetic in a significant percentage of patients. That stresses the importance of the molecular investigation in those cases formerly classified as idiopathic.

## INTRODUCTION

In developed countries, it is estimated that 60% of the cases of prelingual deafness have genetic causes, and the remaining 40% have the most varied etiologies (Marazita et al., 1993, Mustafa et al., 2001). Moreover, 1/1000 children become deaf after learning to speak, before coming of adult age; between 30-50 years of age, 0.3% of the population report hearing loss above 65 decibels (dB) and 2.3% of the population have it between 60 and 70 years of age. The prevalence keeps growing and continues to increase, reaching 50% in 80 year old patients (Paparella et al., 1989; Morton et al., 1991).

In Brazil, environmental factors (67%) are among the major causes of hearing loss, mostly congenital rubella and neonatal anoxia (33.5%), followed by those of unknown etiology (18.5%) and those of recessive autosomal heritage (15.5%). With the major environmental factors, high deaf risk neonates are those with a 2-5% chance of developing hearing loss if they suffer one or more of the following factors: asphyxia or anoxia, with Apgar score below 7,1; bacterial meningitis, specially caused by Haemophilus influenza; prenatal congenital infections (syphilis, toxoplasmosis, rubella, cytomegalovirus, herpes); head and neck malformations; high bilirubin levels (levels that require transfusion); family history of hearing loss; neonatal ICU stay for more than 48 hours; birth weight below 1.5 kilogram and use of ototoxic drugs by the pregnant mother or during the neonatal period (Simões et al., 1992).

Cases of late hearing loss, that being, postlingual, may also occur after exposure to ototoxic drugs, family history of hearing loss, and in some cases it may be a sudden hearing loss of unknown origin, thus being considered idiopathic.

Although genetic therapy is still far from practical use, cochlear implants are a feasible resource, and one available for bilateral severe/or profound hearing loss for those who do not benefit from individual sound amplification devices (hearing aids).

In those cases of patients who lost hearing before learning to speak (prelingual), age is one of the most important factors. In very young patients, results are better than in older children. Usually the cause of this early hearing loss is congenital, of genetic origin, without physical injury to the hearing apparatus, and this favors the cochlear implant.

The 35delG mutation on the connexin 26 gene is not rare; and quite the contrary, its presence in heterozygosis may be found in up to 3% of the individuals of some populations. Notwithstanding, in Italy, its prevalence is around 1:32, in Portugal it is of 1:40, and in Spain it is 1:45. If we group together these three European populations, of which a great part of the Brazilian population descend, we can see that the average frequence of heterozygosis for the 35delG mutation is of 1:42; considering a random union of heterozygosis and the 25% chance of affected descendents, we know that in these regions 1 in 5,069 children would be born deaf because of the 35deIG mutation homozygosis (Gasparini et al., 2000).

Recent progresses in molecular biology, with the finding of different genes involved in hearing loss, allow the possibility of identifying the hearing loss etiology. The high prevalence of mutations on the GJB2 gene and the ease of study allow the diagnosis of many a patient, and have suggested that these individuals would be potential candidates to receive a cochlear implant.

The most frequent mutation in this gene is the one called 35delG, the one most involved in cases of hearing loss of genetic etiology.

This mutation is the loss of a guanine base in the DNA sequence of the gene on position 35. It is very important to search for the 35delG mutation in the hearing loss etiology, since 2 to 4% of the individuals are carriers of this mutation, in other words, they are heterozygotes. The 35delG mutation corresponds from 75 to 80% of the possible mutations found in this gene (Cohn, et al., 1999a; Cohn et al., 1999b).

It is necessary for the individual to inherit two mutated genes, one from the father and the other from the mother, for deafness to set in. This way, it is impossible for connexin 26 to be codified by the altered GJB2 gene. When the patient presents a 35delG mutation in heterozygosis, it means that there is mutation in only one of the alleles, and thus it is possible for the other allele to codify the protein. This implies in a smaller number of codified connexin 26.

The connexin 26 protein is indispensable to the normal functioning of the inner ear, and the gene alterations responsible for its coding is the main cause of hereditary, non-syndromical prelingual deafness, identified by the 35delG mutation study through the “genetic hearing loss test”.

Some studies have concluded that the cochlear implant in patients with GJB2 gene-related hearing loss allows them to acquire voice recognition equal to or better when compared to prelingual deaf children, with deafness of unclear origin, or even with congenital hearing loss, such as those caused by cytomegalovirus (Sinnathuray et al., 2004; Green et al., 2002). This is so, because when the damage occurs to the gene, its expression does not interfere in the placement of the implant, in other words, the cochlea is not involved, such is the case of traumatic or infectious hearing loss, from meningitis or ototoxicity, which damages the cochlear physical structure, often times preventing a larger number of electrodes to be inserted because of cochlear calcification, for instance. Genetic damage happens to the connexons structure, impairing intercellular communication, and this does not harm the cochlear physical structure, favoring the implant.

The feasibility and the benefits of tracking the connexin 26 gene mutations are being reflected on public health. The use of molecular tests, together with audiometric tests will help in the early detection of hearing loss, which is extremely important in managing these patients, specially in cases of progressive hearing loss, since language development in its critical period makes the children learn to communicate before the hearing loss becomes more severe. Moreover, today it is possible to make a predictive diagnosis in those individuals affected by mutations in the connexin 26 gene, even before hearing loss sets in. The consequences of this prediction in both the social and family environment are deafness prevention or to help reduce costs assigned to the special education of such individuals, its medical management and professional decision (Sobe et al., 2000; Sartorato et al., 2000).

The goal of the present work was to check the incidence of 35delG mutation in children in need of and who received cochlear implants and had their hearing loss diagnosed as supposedly idiopathic.

## MATERIALS AND METHODS

This study was carried out in the otorhinolaryngology outpatient ward of the University Hospital - UNICAMP-SP, cochlear implant and human genetics lab departments CBMEG, UNICAMP-SP.

32 children candidates to cochlear implantation, with sensorineural, severe to profound, supposedly idiopathic, bilateral hearing loss. The major inclusion criteria of these children in the cochlear implant department are the non-benefit of individual sound amplification device (hearing aids). These individuals were submitted to a multiprofessional assessment, including otorhinolaryngologists, speech therapists, psychologists, social workers, nurses and geneticists.

The 35delG mutation tracking was carried out based on peripheral blood DNA colected in a purple lid Vacutainer. In order to detect the 35delG mutation we used the allele specific PCR method (AS-PCR), using primers and the aforementioned polymerase chain reaction - modification patented by CBMEG - UNICAMP. (Oliveira et al., 2002). This method easily differentiates the normal allele from the mutant and through two reactions it is possible to distinguish normal homozygotes, 35delG homozygote and heterozygote carriers of the 35delG mutation.

The molecular analysis results were correlated with the hearing loss etiology presented by the patients analyzed.

## RESULTS

In the present study, 32 children with idiopathic hearing loss underwent GJB2 gene 35delG mutation tracking. The mutation was found in 4 homozygotes of the total children assessed (12% of the cases), this being the hearing loss cause. The mutation was found in heterozygosis in 6 children (19% of the cases); It was not possible to diagnose the hearing loss in these patients. The 35delG heterozygosis mutation does not diagnose the hearing loss cause, it only proves the patient is a carrier of the mutation and, as shown in the literature, heterozygote patients can always hear. In the graph below, we can see the results obtained from the genetic study.

## DISCUSSION

With the progress of research in this area, it is clear the importance of gjb2 gene mutation studies due to the easy detection of mutations in the conexine 26, specially mutation on 35delg - considered the most frequent mutation of any gene already studied in caucasoids. This is the first gene indicated for molecular analysis in families with sensorineural hearing loss (sobe et al., 2000).

**Chart 1:**
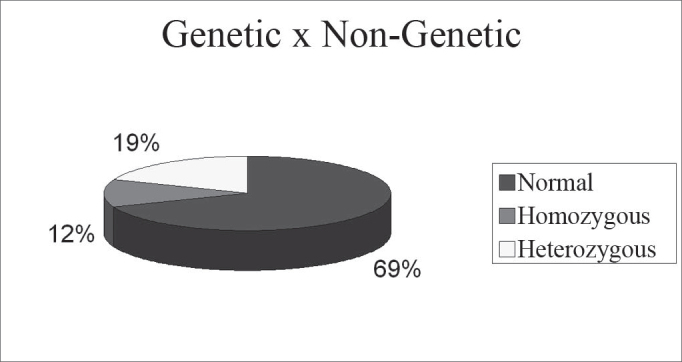

In a prior study carried out in a sample of the Brazilian population, mutations to the GJB2 gene were found in 22% of the families with non-syndromical sensorineural hearing loss, once again pointing out that molecular analysis of this gene in patients with non-syndromical hearing loss should be the first step in determining hearing loss causes in our country (Oliveira et al., 2001). This is particularly true for cases of family hearing loss, among which the frequence of mutations in this gene was found to be of 50%, but also for sporadic cases, among which the frequence was a bit higher than 11% (approximately 1:9) (Oliveira et al., 2002). In Brazil, the prevalence of 0.97% of 35delG mutation carriers was found to be approximately 1:103 heterozigotes, in a survey carried out with 620 newborns in the region of Campinas-SP (Oliveira et al., 2004).

Our results are in agreement with studies carried out and described in the literature for different populations. The relative distribution of the 35delG mutation for non-syndromical hearing loss in these populations varied from 0% (Oma, Korea and Japan) to 70% (Italy, Spain, Greece), showing the genetic heterogeneity among the different countries, despite the fact that some of these studies were based on a small number of patients and the investigation criteria regarding mutation tracking methods were different as well.

## CONCLUSION

Data obtained in the present study confirmed the high prevalence of GJB2 gene 35delG mutation in cases of profound and bilateral non-syndromical sensorineural hearing loss. It is also possible to use genetics to diagnose the cause of hearing loss in a significant number of children.

The results stress the importance of molecular studies in patients with supposedly idiopathic hearing loss, since this test allows us to clarify the hearing loss etiology when there is a positive result.
